# Evolutionary analysis of slope direction deformation in the Gaojiawan landslide based on time-series InSAR and Kalman filtering

**DOI:** 10.1371/journal.pone.0316100

**Published:** 2024-12-31

**Authors:** Jingchuan Yao, Runqing Zhan, Jiliang Guo, Wei Wang, Muce Yuan, Guangyu Li, Bo Zhang, Rui Zhang

**Affiliations:** 1 China Academy of Railway Sciences Corporation Limited, Beijing, China; 2 China Railway Desigin Corporation, Tianjin, China; 3 Faculty of Geosciences and Engineering, Southwest Jiaotong University, Chengdu, China; 4 Tieke Inspection Corporation Limited, Beijing, China; 5 Railway Engineering Research Institute, China Academy of Railway Sciences Corporation Limited, Beijing, China; 6 Institute of Mountain Hazards and Environment (IMHE), Chinese Academy of Sciences, Chengdu, China; Universiti Kebangsaan Malaysia, MALAYSIA

## Abstract

The existing landslide monitoring methods are unable to accurately reflect the true deformation of the landslide body, and the use of a single SAR satellite, affected by its revisit cycle, still suffers from the limitation of insufficient temporal resolution for landslide monitoring. Therefore, this paper proposes a method for the dynamic reconstruction and evolutionary characteristic analysis of the Gaojiawan landslide’s along-slope deformation based on ascending and descending orbit time-series InSAR observations using Kalman filtering. Initially, the method employs a gridded selection approach during the InSAR time-series processing, filtering coherent points based on the standard deviation of residual phases, thereby ensuring the density and quality of the extracted coherent points. Subsequently, the combination of ascending and descending orbit data converts the landslide’s line of sight (LOS) deformation into along-slope deformation. Finally, the Kalman filtering method is utilized for dynamic reconstruction of the landslide deformation, and an analysis of the evolutionary characteristics of the landslide is conducted to explore its impact on transportation infrastructure, thereby significantly improving the temporal resolution and accuracy of landslide monitoring. To verify the feasibility of the algorithm, this paper selects the Gaojiawan landslide as a typical study area. Based on the ascending and descending Sentinel-1 SAR data from 2016 to 2023, it extracts the temporal series of slope body deformation to further explore its impact on the internal transportation infrastructure of the slope body. Experimental results show that the combination of ascending and descending SAR data and Kalman filtering has improved the time resolution of landslide monitoring to six days. It was found that two significant slips occurred in the slope body in January 2016 and June 2021, while other periods were relatively stable. Further discussion and analysis reveal that there is a difference in the slip deformation rate between the upper and lower parts of the slope body, and the shear stress caused by dislocation deformation.

## Introduction

Landslides, as a highly common and significantly hazardous geological disaster [1–5], are widely distributed across the globe [6], causing not only substantial economic losses but also posing serious threats to human safety [7]. In recent years, geological disasters have occurred frequently in China, with landslides accounting for more than 70% of the total number of disasters. Particularly, the western regions of China have become one of the high-risk areas for frequent landslide occurrences [8, 9]. In January 2016, bulging cracks appeared on the surface about 100 meters north of the central front edge of the Gaojiawan landslide. The lining structure of a tunnel, which passed underneath the lower part of the landslide body, was severely damaged. Approximately 600 meters of the tunnel shifted, forcing the suspension of its operation. Therefore, conducting dynamic reconstruction and analysis of the evolution characteristics of the Gaojiawan landslide is of great scientific value and practical significance for ensuring the safety of tunnel operations and improving disaster early warning capabilities [10].

Traditional landslide disaster monitoring primarily relies on methods such as leveling instruments, total stations, and Global Positioning System (GPS) technology. These technologies are based on the observation of discrete point targets, leading to limited monitoring scope, high costs, and especially in complex mountainous environments, questionable safety. In recent years, Interferometric Synthetic Aperture Radar (InSAR) technology, with its advantages of large observational coverage, high measurement precision, and high spatial resolution, has become an indispensable tool for landslide disaster monitoring [11–13]. Early InSAR landslide monitoring efforts were limited to using a small amount of SAR imagery for Differential InSAR (DInSAR) detection of landslide activity. Although DInSAR can effectively detect minor surface deformations, its monitoring accuracy is susceptible to seasonal variations in vegetation cover and cannot effectively correct delay errors of radar waves during atmospheric transmission [14]. To overcome these limitations of DInSAR, research on landslide deformation detection methods based on multi-temporal satellite SAR imagery has been conducted by numerous experts and scholars domestically and internationally, proposing a series of corresponding time-series InSAR methods [15–21], including: Persistent Scatterer InSAR (PSI), PS Pair Interferometry (PSP), Interferometric Point Target Analysis (IPTA), SqueeSAR, Joint-Scatterer InSAR (JSInSAR), The Stanford Method for PS (StaMPS), Spatiotemporal Unwrapping Network (STUN), and others. Methods utilizing multiple master images include the Small Baseline Subset (SBAS), Stable Point Network (SPN), and Temporarily Coherent Point InSAR (TCP-InSAR). In addition to the common multi-temporal InSAR methods, Blind Source Separation (BSS) and waveform coding techniques have also been proposed in SAR systems. As effective methods for de-blurring and signal processing, they can play an important role in improving the quality and accuracy of SAR data, especially in landslide monitoring in complex terrain, where they have potential applications [22–24].

The methods based on phase information from multi-temporal SAR imagery, which are processed through interferometry, are typically suited for extracting the time-series displacement of the landslide surface in a slow, steady state [25, 26]. This is because the phase unwrapping process in SAR interferometry requires the interferometric phases to be continuous in both time and spatial scales. However, single-platform or single-orbit satellite-based InSAR monitoring can only acquire one-dimensional deformation information along the Line of Sight (LOS) of the landslide body, failing to directly capture deformation information consistent with the direction of landslide movement [27]. In high mountain and canyon areas, due to significant terrain slopes and varying slope directions, and influenced by satellite imaging attitudes and local incidence angles, InSAR monitoring results from different viewpoints can vary significantly [28]. This poses challenges to the interpretation of InSAR results and is not conducive to understanding the mechanisms and dynamics of the deformation process [29, 30]. To address these issues, researchers have conducted a series of three-dimensional decomposition studies on landslide deformations in complex regions [28, 30, 31]. Since the landslide body slides obliquely down along a weak slope of a hillside, the common method of three-dimensional monitoring involves decomposing the deformation information into north-south, east-west, and vertical directions [32, 33]. Therefore, this simple three-dimensional decomposition may not accurately reflect the magnitude of the landslide deformation. Investigating the deformation information along the downslope direction is crucial for revealing the laws of landslide movement.

The deformation results obtained from the three-dimensional decomposition of InSAR data are still subject to the limitation of long revisiting cycles of satellite platforms, typically around 11 to 24 days. This limitation leads to a lower temporal resolution in landslide monitoring, making it challenging to achieve long-term dynamic monitoring [34]. With the incorporation of new data from different platforms/orbits, the temporal resolution of landslide monitoring can be further enhanced through curve fitting methods [35]. In 1960, R.E. Kalman introduced a new method for linear filtering and prediction, which outlined a recursive solution to the problem of discrete data linear filtering, known as Kalman filtering [36]. Kalman filtering is primarily used in two aspects: firstly, for the optimal estimation of quantities of interest that cannot be directly measured but can be indirectly measured; and secondly, for estimating system states by combining various sensor measurements, which may be influenced by noise [37]. Its main advantage is extracting the most accurate or relatively precise data from multiple uncertain data sources. With the rapid development of computer hardware and software and significant advancements in computing power, Kalman filtering, as a method for optimal estimation of dynamic data states, has been widely applied in various fields such as navigation, communications, aerospace, and computer vision [19, 37, 38].

Although InSAR technology has made significant progress in landslide monitoring, how to combine multi-orbit SAR data to improve the temporal resolution of landslide monitoring while obtaining movement characteristics that are more consistent with the actual behavior of landslides remains a pressing issue to be addressed. Studies have shown that combining ascending and descending orbit data with the Kalman filtering method can significantly improve the temporal resolution and accuracy of landslide monitoring. Therefore, this paper focuses on the Gaojiawan landslide as the study area. Based on InSAR technology to acquire surface deformation information of the landslide area, this study converts the LOS deformation of the landslide to the slope direction by combining ascending and descending orbit SAR data. Utilizing the Kalman filtering method for the dynamic reconstruction of landslide deformation, this research aims to refine the monitoring and assessment system for landslide disasters, providing a scientific basis for disaster prevention and mitigation.

## Study area and data sources

**Fig 1:**
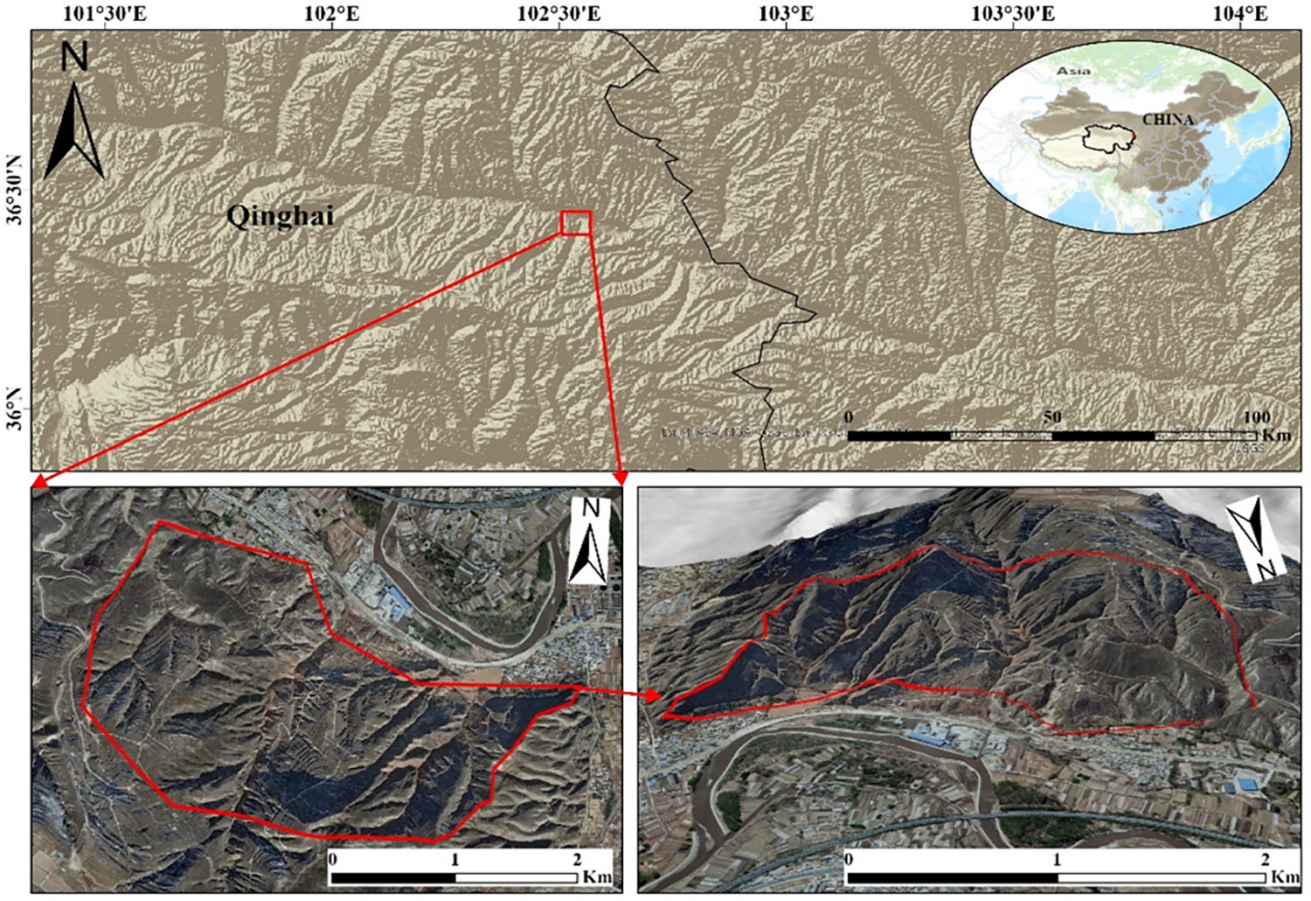
Geographic location of the study area. Image basemaps and topography appearing in the text are from ESRI (www.ESRI.com).

Landslide disasters are frequent and prevalent in the loess regions of Northwest China. This research is focused on the Gaojiawan landslide area on the right bank of the Huangshui River from Hexi Village to Shuangta Village in Donghongshui Town, Ledu District, Haidong City, Qinghai Province. As depicted in Fig 1, this area is situated on the southern bank of the Huangshui River in a hilly region, with geographic coordinates extending from 102°30’00" to 102°33’45" east longitude and from 36°25’00" to 36°27’43" north latitude, and is 15.0 km away from the district’s government seat. The terrain of the area is complex, located in the loess hilly and gully region of the Loess Plateau, with a general topography that slopes down from south to north. The highest point is at an elevation of 2466 meters, and the lowest point is at 1903 meters, with a relative elevation difference of 563 meters [39].

**Fig 2:**
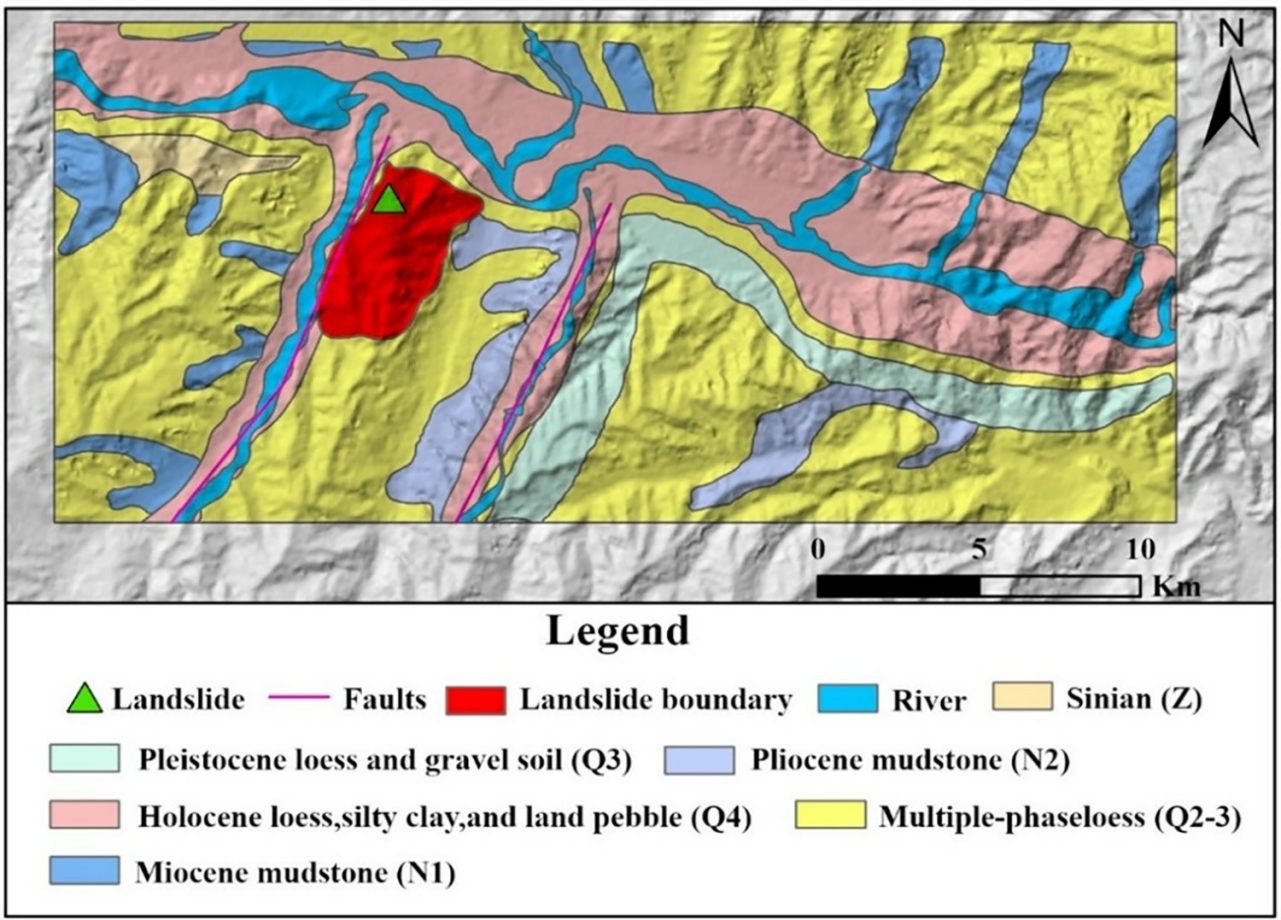
Lithology of the study area.

The Gaojiawan landslide area experiences a plateau temperate semi-arid climate, characterized by low annual precipitation and high evaporation rates, long freezing periods, and short frost-free periods, along with large diurnal temperature variations [40]. Precipitation is unevenly distributed throughout the year, primarily concentrated from May to September, accounting for 87.4% of the annual precipitation, which is also the period prone to geological disasters. Precipitation is one of the leading factors inducing landslides, mudslides, and other geological disasters in the area vv. The region is located at the junction of the Qinghai-Tibet Plate and the Qilian Mountain fold belt, squeezed between the Laji Mountain and the Daban Mountain, belonging to an area of intense neotectonic movement and seismic activity. The area is riddled with large and small faults, resulting in poor stability of the regional slope bodies [41]. As shown in Fig 2, the stratigraphy of the Gaojiawan landslide area is primarily Quaternary loess and Paleogene mudstone, mainly composed of weathered rock debris, clay, and organic matter, forming complex multi-phase large to gigantic landslides [42]. The strata have a fine particle size, usually appearing in grey, brown, or black, characterized by high water content and great plasticity, conducive to landslide formation. It has been recorded that since 2012, tensile cracks have repeatedly appeared at the rear of the Gaojiawan landslide mass, with significant deformation events occurring in 2016 and 2021 [43]. The frequent geological disaster activities have severely impacted the safety of people’s lives and property and the health of infrastructure, particularly posing a significant hidden danger to the safety of major transportation infrastructure operations [44]. National Highway 109 Gansu-Qinghai, G6 Beijing-Tibet Expressway, and the Lanzhou-Xinjiang Railway all cross through the study area, with landslide occurrences posing a severe threat to the safe operation of these major transport routes [45]. Therefore, the purpose of this study is to provide reference information for disaster prevention and reduction efforts through monitoring and analysis of the Gaojiawan landslide area.

The C-band Sentinel-1 data covering the Gaogiawan landslide area from 2016 to 2023 were collected for the experiment. Among them, the ascending orbit data were 208 views and the descending orbit data were 170 views. The spatial resolution of Sentinel-1 was 5m×20m with a width of 250 km, and the digital elevation model (DEM) used was the 30m-resolution STRM1 data, while the precision orbital data files provided by ESA were used for the refinement of the spatial baseline.

## Methods

**Fig 3:**
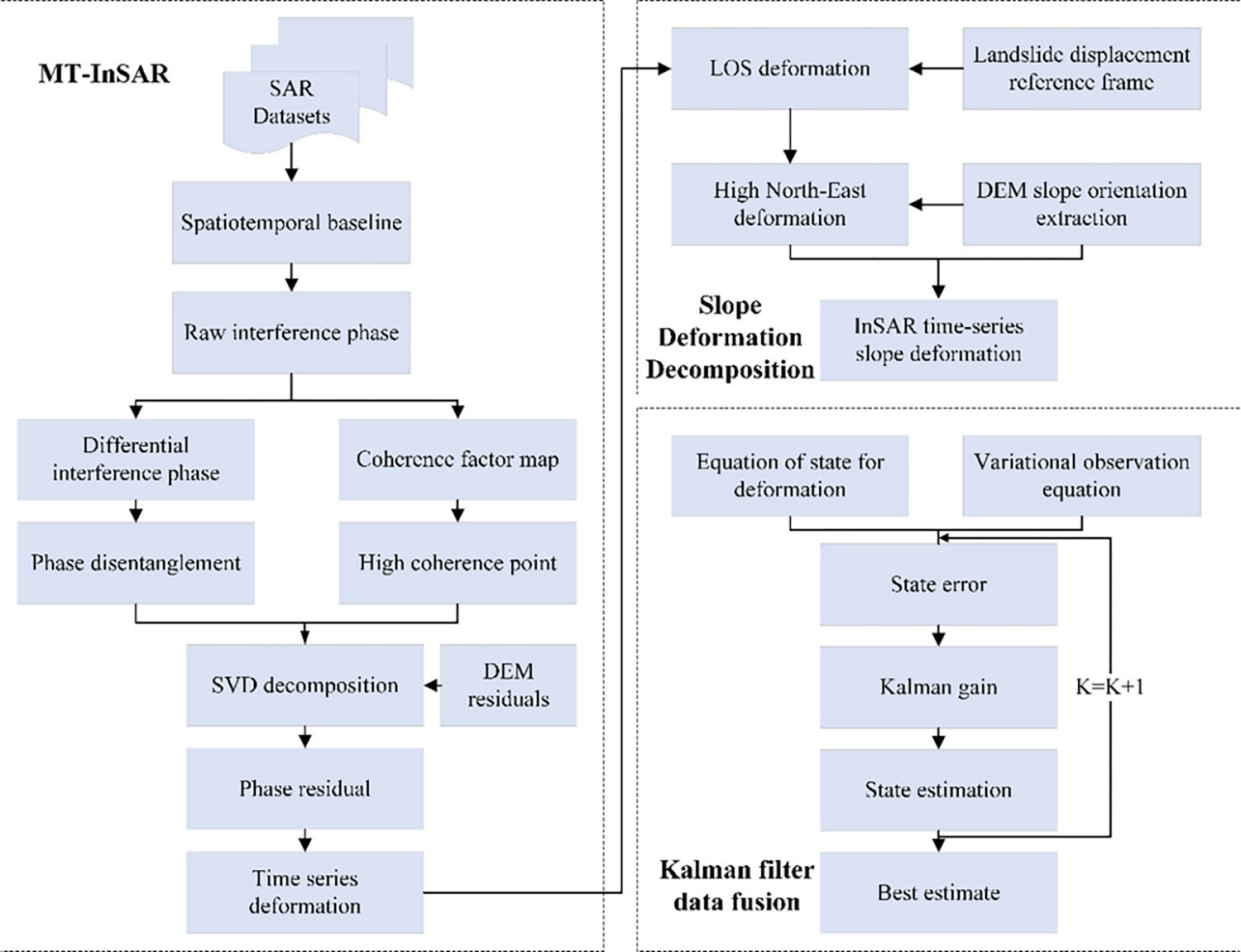
General technical flow chart.

This study builds a Kalman filter dynamic reconstruction model of along-slope deformation based on the ascending and descending orbit timing sequence InSAR, which is mainly divided into the following three steps. First, the time-series deformation of the study area is obtained based on Multiple Temporal InSAR (MT-InSAR) technology; second, the establishment of a three-dimensional landslide displacement coordinate system and the transformation of time-series slope deformation; third, the Kalman filter fusion of the slope deformation of the lifting rail. The main technical process is shown in Fig 3.

### MT-InSAR landslide monitoring method

The MT-InSAR technique is based on short-time baseline thresholding for the free combination of multiple image pairs, and then select the interferometric pairs with high coherence to do differential interferometric processing, based on which the linear deformation is obtained based on the SVD decomposition, and finally the deformation model is used to obtain the DEM residuals, and the spatial-domain filtering and temporal-domain filtering are used to obtain the nonlinear deformation and the atmospheric phase delay.

Assume that a total of N+1 SAR images are acquired in the study area, select one image as the main image, register and resample the remaining N SAR images to the coordinate system of the main image, and select the matching image among all free combination differential interference pairs. The interference pairs of the time baseline and the spatial baseline threshold are used to obtain M-amplitude differential interference patterns. then there is:

$$\frac{N+1}{2}\leq M\leq N(\frac{N+1}{2})$$ (1)

Taking the initial time *t*_0_ as the reference time, the differential phase *ϕ*(*t*_*i*_) at any time *t*_*i*_(*i* = 1,2,…,*N*) relative to time *t*_0_ is an unknown quantity, and the differential interference phase *δϕ*(*t*_*k*_)(*k* = 1,2,…*M*) obtained during the data processing process is an observed quantity. Then the phase value of pixel (*r*,*c*) in the i-th (*i* = 1,2,…*M*) differential interference image is:

$$\delta \varphi_{i}(r,c)=\varphi (t_{A},r,c)-\varphi (t_{B},r,c)\approx \frac{4\pi }{\lambda }[d(t_{A},r,c)-d(t_{B},r,c)]$$ (2)

In the formula, *λ* is the radar wavelength; *d*(*t*_*A*_,*r*,*c*) and *d*(*t*_*B*_,*r*,*c*) are the deformation displacement of pixel (*r*,*c*) along the radar line of sight direction at time *t*_*A*_ and *t*_*B*_ respectively.

For all the obtained interferograms, a linear equation of the differential interference phase between the deformation variables at the time of image acquisition is constructed, including M equations with N unknowns, as follows:

δ=Aφ (3)

In the formula, *δ* represents the matrix composed of the unwrapped phase of the interference pair, *φ* represents the parameter matrix, and A is the coefficient matrix.

If all interference pairs belong to the same sub-baseline set, then the rank of matrix A is *N*(*M*≥*N*), and its least squares solution is:

$$\hat{\varphi }={\left(A^{T}A\right)}^{-1}A^{T}\delta \varphi$$ (4)

If A contains multiple sub-baseline sets, it is necessary to perform singular value decomposition on it to solve for the phase change rate v in each time period, and then calculate and restore the phase time series based on this, and then obtain the deformation time series.

Traditional MT-InSAR technology primarily relies on the coherence coefficient for the extraction of coherent points, and builds phase models and calculates deformation parameters based on this. During the process of identifying coherent points using traditional algorithms, it is necessary to balance two factors: the window size used for calculating the coherence coefficient and the validity of the results. As a result, there is a risk of either missing or over-selecting points. The grid-based point selection method, on the other hand, initially selects pixel points according to a regularly distributed spatial grid, that is, by setting the number of extractions and the interval in the range and azimuth directions to generate coherent points (CS), thereby allowing for preliminary adjustment of point density as needed. Since MT-InSAR technology limits the size of the spatial-temporal baseline when forming interferometric pairs, it is feasible to calculate for these points that have not been pre-screened with conditions. At the same time, to ensure the validity of the results, further selection of points is needed during the subsequent deformation calculation process based on the size of the residual phase standard deviation for each target point, in order to eliminate those of lower quality. This method not only simplifies the calculation process but also, because the processing is performed on individual pixels, effectively extracts coherent points surrounded by noisy pixels, which is not possible with traditional window-based coherence coefficient calculations. Coupled with appropriately relaxed selection criteria, the density of deformation points in the results is increased.

### Landslide InSAR aspect projection conversion model

This section may be divided by subheadings. It should provide a concise and precise description of the experimental results, their interpretation, as well as the experimental conclusions that can be drawn.

The general laws of landslide movement show that landslides move downward along the sliding surface under the action of gravity, so the geometry of the sliding surface plays a decisive role in the material migration of the landslide, and its slope and slope direction largely affect the behavior of the landslide. Strength and direction.

The side-view imaging geometric frame of the sensor platform can establish a unified coordinate reference datum for the ground observation target P, and measure the true displacement of the target in three-dimensional space. During the SAR interferometry process, the LOS deformation result can be expressed as the sum of the projections of the three-dimensional components of the ground target point P in the LOS upward direction, that is:

$$D_{LOS}=D_{Z}\cdot cos\theta +D_{N}\cdot sin\varphi sin\theta -D_{E}\cdot cos\varphi sin\theta$$ (5)

In the formula, *D*_*LOS*_ is the deformation in the LOS direction, *D*_*Z*_, *D*_*N*_ and *D*_*E*_ are the deformation components of the target point in the high, north, and east directions respectively, *φ* is the satellite heading angle, and *θ* is the local incident angle of the radar signal.

**Fig 4:**
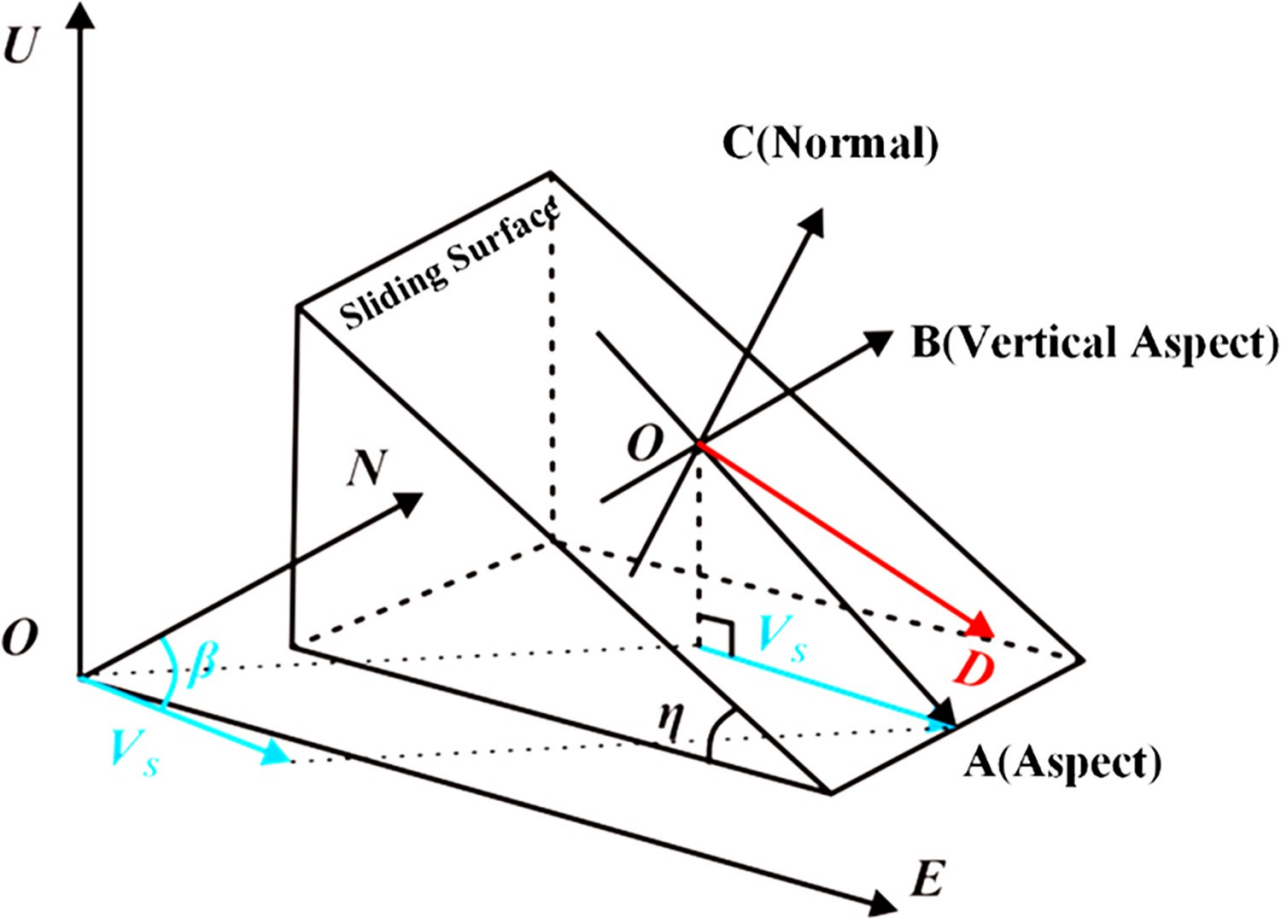
LOS decomposed into three dimensions.

According to the relationship between the radar line-of-sight deformation and the three-dimensional deformation component of the station center rectangular coordinate system provided in Fig 4:

$$D_{LOS}=\left[\begin{array}{ccc} -cos\mu sin\theta & sin\mu sin\theta & cos\theta \end{array}\right][\begin{array}{c} D_{E}\\ D_{N}\\ D_{U} \end{array}]-K$$ (6)

In the equation, *θ* and *μ* represent the radar wave’s side-looking angle and the radar satellite’s heading angle, respectively. The systematic deviation between the InSAR-calculated LOS deformation velocity R and the true radar LOS deformation velocity D for each target point is a constant. Through equation, the relationship between the radar LOS deformation amount and the three-dimensional deformation components of the sliding surface can be derived:

$$D_{LOS}=\left[\begin{array}{ccc} a & b & c \end{array}\right][\begin{array}{c} D_{A}\\ D_{B}\\ D_{C} \end{array}]-K$$ (7)

Among them, A, B, and C represent the projection coefficients of the deformation observation along the slope, vertical slope, and normal directions respectively. The specific expressions are:

$$\left[\begin{array}{ccc} a & b & c \end{array}\right]={\left[\begin{array}{c} -cos\mu sin\theta cos\eta sin\beta +sin\mu sin\theta cos\eta cos\beta -sin\eta cos\theta \\ cos\mu sin\theta cos\beta +sin\mu sin\theta sin\beta \\ -cos\mu sin\theta sin\eta sin\beta +sin\mu sin\theta sin\eta cos\beta +cos\theta cos\eta \end{array}\right]}^{T}$$ (8)

In order to enhance the temporal resolution of landslide monitoring as much as possible, we assume that each element on the landslide surface moves solely along the direction of the landslide surface slope downward. Based on this characteristic of the landslide movement mechanism, we simplify the model by assuming that deformations in both the vertical slope direction and the normal direction to the slope surface are zero. With this movement model, it is possible to estimate the slope-wise deformation of a landslide using a single track of InSAR. The above can be simplified and represented as follows:

$$D_{A}=a^{-1}D_{LOS}+K$$ (9)

In the equation, *a* represents the projection coefficient matrix, which is determined by the orbital angles of various satellite platforms, the satellite incidence angles, the slope and aspect of the landslide surface pixels, and varies at different points on the landslide surface. Its expression is as follows:

$$a={\left[a_{1},a_{2},\cdots ,a_{n}\right]}^{T}$$ (10)

In Eq (9), *K* denotes the orbital system bias:

$$K={\left[K_{1},K_{2},\cdots ,K_{n}\right]}^{T}$$ (11)

The final calculation yields the slope-wise deformation values for each pixel (*i*,*j*) in orbit N, and the corresponding landslide displacement time series *S*^*N*^ is determined, where n represents the number of monitoring time nodes for orbit N.


$$S^{N}=[S_{1}^{N},S_{2}^{N},\ldots ,S_{n}^{N}]T$$ (12)

### Ascending and descending orbit time-series InSAR Kalman filter fusion model

The key to Kalman filtering is constructing the state equation and the observation equation. For a specific ground point on a landslide surface, at the monitoring time *k*, its displacement *S*_*k*_ and movement velocity *v*_*k*_ can be expressed as:

$$\left\{ \begin{array}{l} S_{k}=S_{k-1}+v_{k-1}\Delta t+\frac{u\Delta t^{2}}{2}\\ v_{k}=v_{k-1}+u\Delta t \end{array}\right.$$ (13)

The state at monitoring time *k* can be represented by a matrix as:

$$[\begin{array}{c} S_{k}\\ v_{k} \end{array}]=[\begin{array}{cc} 1 & \Delta t\\ 0 & 1 \end{array}][\begin{array}{c} S_{k-1}\\ v_{k-1} \end{array}]+[\begin{array}{c} \frac{\Delta t^{2}}{2}\\ \Delta t \end{array}]u$$ (14)

In the formula, *S*_*k*_, *v*_*k*_, *S*_*k*−1_, and *v*_*k*−1_ represent the displacement values and velocities at the monitoring event nodes *k* and *k*−1, respectively. The state at time *k* is an estimated value predicted based on the time *k*−1; *u* represents the acceleration over the time interval Δ*t* between the two monitoring events.

For the entire deformation cycle of a landslide mass, it is only when the landslide enters an accelerated deformation stage under external influences that we observe *u*>0, and this value gradually increases as the landslide motion intensifies, reaching a maximum at the critical moment of impending failure. However, for the majority of the time, the landslide mass remains in a stage of no deformation or uniform deformation, during which the deformation acceleration *u* is almost negligible. Therefore, we consider the acceleration variable of the landslide mass within the monitoring interval as prediction state noise, which is then corrected by the observed value at the subsequent moment. Thus, the Kalman filtering equations can be represented as follows:

$$\left\{ \begin{array}{l} {\hat{X}}_{\left(k|k-1\right)}=F{\hat{X}}_{\left(k-1\right)}\\ P_{\left(k|k-1\right)}=FP_{\left(k|k-1\right)}F^{T}+Q\\ G_{\left(k\right)}=P_{\left(k|k-1\right)}H^{T}[HP_{\left(k|k-1\right)}H^{T}+R]-1\\ {\hat{X}}_{\left(k\right)}={\hat{X}}_{\left(k|k-1\right)}+G_{\left(k\right)}[Z_{\left(k\right)}-H{\hat{X}}_{\left(k|k-1\right)}]\\ P_{\left(k\right)}=[I-G_{\left(k\right)}H]Pkk-1 \end{array}\right.$$ (15)

In the equation, ${\hat{X}}_{\left(k|k-1\right)}$ represents the predicted value of the state at time *k* based on the state at time *k*−1, ${\hat{X}}_{\left(k-1\right)}$ is the optimal estimate of the state at time *k*−1, *F* is the state transition matrix; *P*_(*k*|*k*−1)_ is the predicted covariance matrix at time *k*−1, *Q* is the process noise of the prediction model; *G*_(*k*)_ is the gain matrix, *H* is the observation matrix, *R* is the observation noise; ${\hat{X}}_{\left(k\right)}$ is the optimal estimate of the state at time *k* after correction, *Z*_(*k*)_ is the actual observed value at time *k*, *P*_(*k*)_ is the updated covariance matrix, and *I* is the identity matrix.

In this paper, the state transition matrix $F=[\begin{array}{cc} 1 & \Delta t\\ 0 & 1 \end{array}]$ is used to estimate the current state from the previous state. If the state transition matrix does not conform to the landslide motion transition model of this paper, the filtering process will diverge rapidly; the prediction model noise covariance is $Q=[\begin{array}{cc} \frac{(u\Delta t^{2}}{2{)}^{2}} & 0\\ 0 & {\left(u\Delta t\right)}^{2} \end{array}]$, and the observation matrix is *H* = [1,0]. The observation noise covariance *R* is derived from the residuals obtained during the deformation calculation of each InSAR slope direction, which characterizes the sensitivity of the system gain. That is, the smaller the observation noise covariance for the corresponding orbit, the faster the system’s corrective transient response, and conversely, the slower it is. Incorporating the observation noise covariance *R* into the calculation of the gain matrix *G* allows for balancing the magnitudes of the prediction state covariance and the observation noise covariance, to judge the degree of deviation between the measured and predicted values, and to continuously update the weight ratio of model predictions to observations. The larger the value of *G*, the greater the influence of the measured values, and the higher the credibility of the measured values; conversely, the predicted model values are trusted more. Moreover, through the gain matrix *G*, the representation of observation residuals can also be transformed from the observation domain to the state domain, facilitating the next iteration of prediction.

Based on the aforementioned Kalman filter optimized autoregressive model, by optimizing the weights of predicted and measured values, more accurate estimates at time k can be extracted from monitoring data collected by multiple different sensors. For landslide deformation monitoring with multiple InSAR monitoring tracks, the dense monitoring event nodes can be achieved through the iterative and corrective multi-source data slope deformation fusion of the Kalman filter in this paper. Ultimately, the total number of monitoring events *T* can be obtained.


$$T=n^{1}+n^{2}+\cdots +n^{N}$$ (16)

In the formula, *n*^1^, *n*^2^, and *n*^*N*^ represent the number of monitoring event nodes for Sensor 1, Sensor 2, and Sensor N, respectively. *T* is the sum of the monitoring event nodes for all tracks. By increasing the number of monitoring event nodes within the same time period, the purpose of improving the temporal resolution of the landslide monitoring is achieved.

## Results

### Deformation results

**Fig 5:**
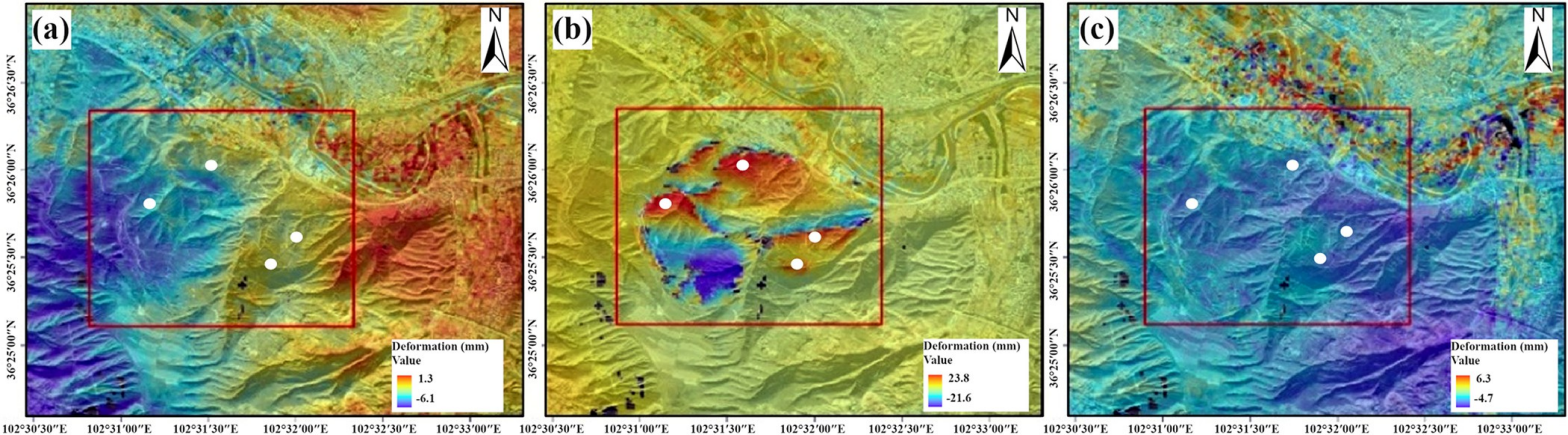
Gaojiawan landslide ascending deformation map. (a) Pre-slip InSAR monitoring results. (b) InSAR monitoring results in sliding. (c) Post-slip InSAR monitoring results.

**Fig 6:**
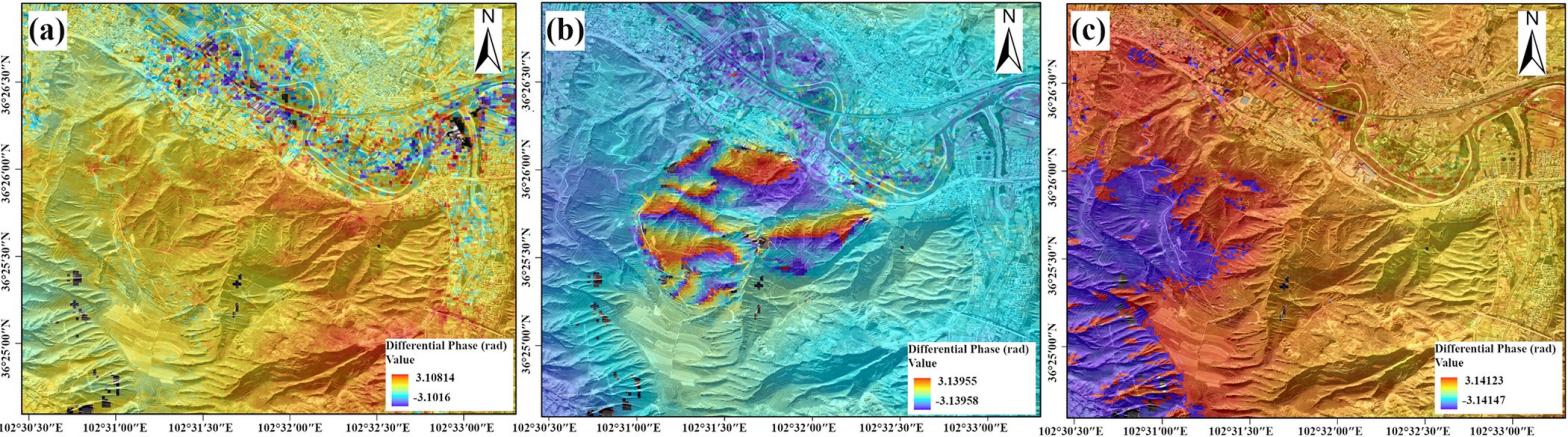
Differential ascending phase map. (a) InSAR differential phase after sliding. (c) InSAR Differential Phase in Slide. (c) InSAR differential phase after sliding.

We conducted differential interferometric processing on the Gaojiawan landslide for the pre-landslide, during-landslide, and post-landslide stages. Fig 5 presents the deformation of the landslide in Gaojiawan Village, Hongshui Town, obtained through the processing of Sentinel-1A ascending images from November 2015 to March 2016. Fig 6 shows the corresponding phase diagram. The analysis indicates that there was significant deformation between January and February 2016 compared to other periods, characterized by notable subsidence in the northeast direction of the mountain, with a slight uplift trend on the southwest side. Specifically, Fig 5A represents the deformation of the landslide body from November 2015 to January 2016, during which the landslide body remained overall stable, with a surface deformation of -5 mm. Fig 5B represents the deformation of the landslide body from January to February 2016, during which significant sliding occurred on the north and east sides of the mountain, with a maximum subsidence of 23.8 mm. The southwest side of the mountain exhibited a slight uplift trend, with an uplift of 10.8 mm and an uplift deformation of 21.6 mm at the bottom. Fig 5C represents the deformation of the landslide body from February to March 2016, during which the landslide body remained generally stable, with an overall deformation rate of 6 mm.

**Fig 7:**
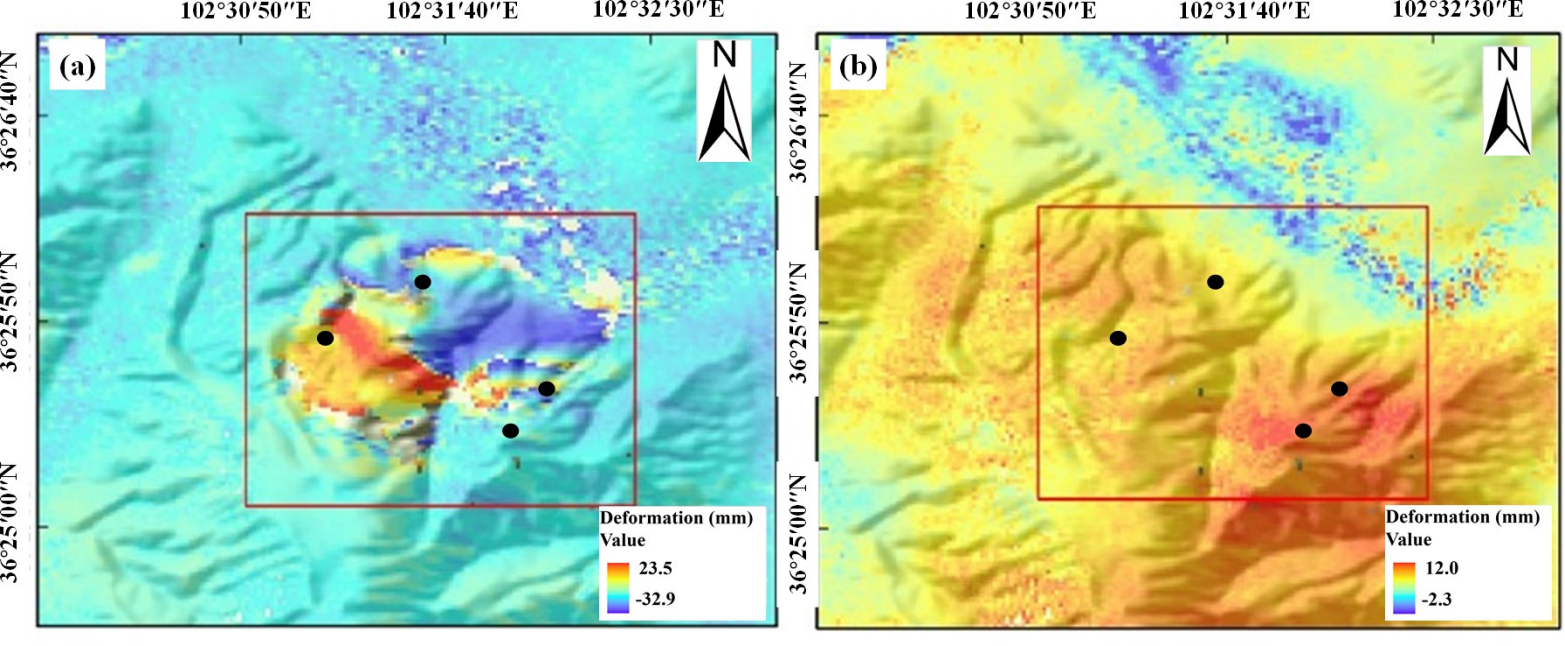
Gaojiawan landslide descending deformation map. (a) December 2015-January 2016 InSAR surface deformation. (b) InSAR surface deformation from January to February 2016.

**Fig 8:**
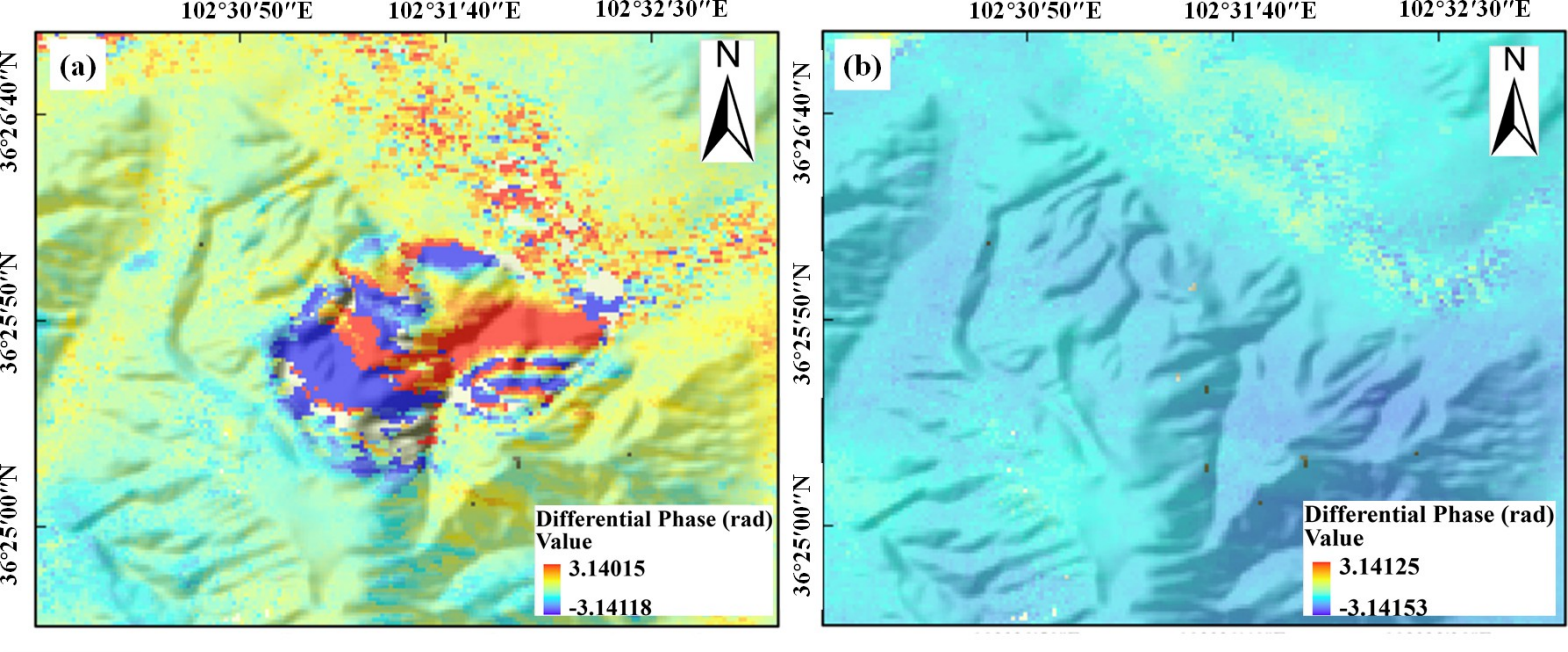
Differential descending phase map. (a) December 2015-January 2016 InSAR differential phase. (b) InSAR differential phase from January to February 2016.

Fig 7 presents the deformation of the landslide in Gaojiawan Village, Hongshui Town, obtained through the processing of Sentinel-1A descending images from December 2015 to February 2016. Due to the limited dataset in the range from November 2015 to March 2016, only two time periods, December 8, 2015, to January 25, 2016, and January 25, 2016, to February 18, 2016, were selected for differential interferometry. Fig 8 shows the corresponding phase diagram. The analysis indicates that significant deformation occurred between December 2015 and January 2016 compared to February 2016, characterized by noticeable sliding at the mountain summit and on both sides of the gullies, with a clear subsidence trend, while the mountain area beneath the gully junction exhibited an upward trend. Specifically, Fig 7A represents the deformation of the landslide body from December 2015 to January 2016, during which the mountain exhibited significant deformation. The south side of the mountain experienced noticeable sliding, with a maximum displacement of 20.79 mm. The north side of the mountain remained stable, with a deformation of 8 mm. Fig 7B represents the deformation of the landslide body from January to February 2016, during which the landslide body remained overall stable, with a surface deformation of -1 mm.

### Kalman-filtered downslope temporal deformation melting

**Fig 9:**
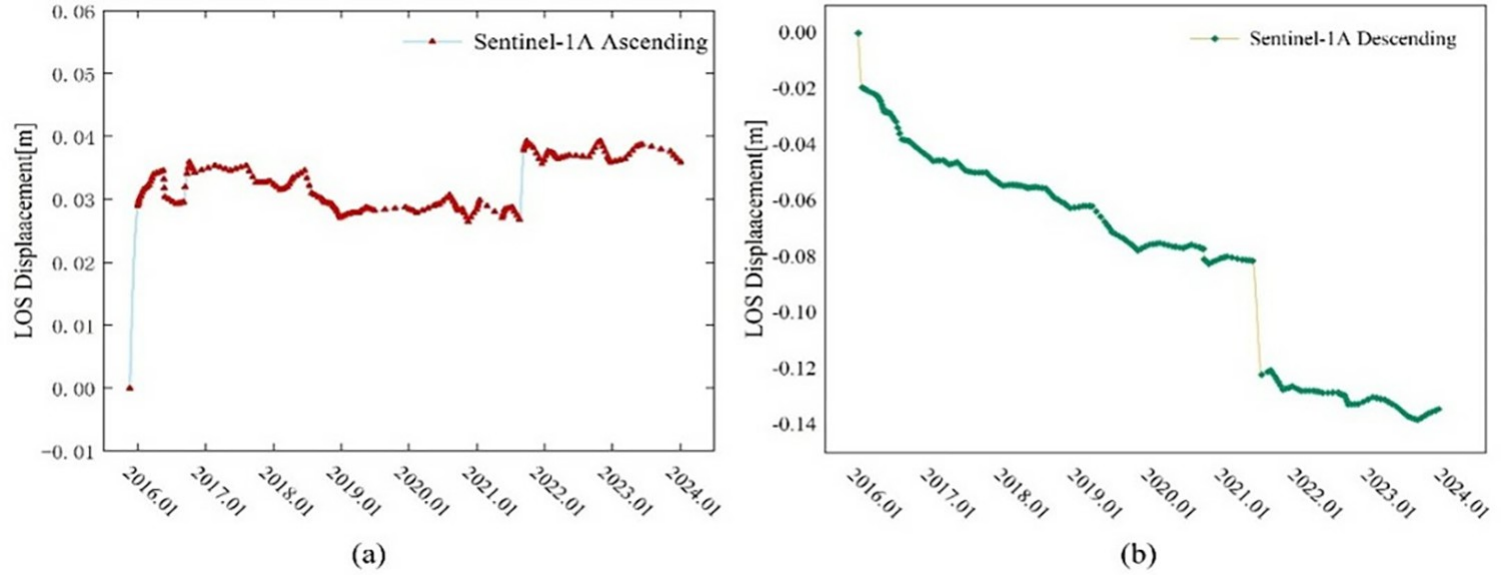
Sentinel-1A ascending/descending orbital LOS displacement time profile at POI point.

To further analyze the spatiotemporal movement characteristics of the landslide during its occurrence, we comprehensively considered the coherence of InSAR results and the displacement characteristics of the landslide body. A point of interest (POI) was selected at the back edge of the landslide, with coordinates of 102.53220E/36.42655N, as the research object. The deformation characteristics along the LOS direction during the ascending and descending orbits were statistically analyzed, as shown in Fig 9.

From the LOS deformation curve at this point, it can be observed that there is persistent displacement after the landslide in 2016. By the end of 2021, the cumulative deformation in the LOS direction of Sentinel-1A ascending and descending orbits reached 2.9 cm and 1.9 cm, respectively. During the second landslide from 2021 to 2022, both the Sentinel-1A ascending and descending orbit time series detected significant abrupt changes, indicating that the slow movement of the landslide body after the first landslide led to the instability of the sliding mass, causing the second landslide. As of January 2022, Sentinel-1A ascending and descending orbits have observed cumulative displacement deformations of 3.8 cm and 13.2 cm, respectively, along the LOS direction of the landslide body. It can be seen that although both ascending and descending orbit observations detected displacement changes during the landslide, differences in radar sensor incidence angles, landslide slope, and satellite heading angles during the observation period led to different LOS displacement components on different satellite platforms. Additionally, differences in terrain relief and line-of-sight angles resulted in significant differences in the sensitivity and accuracy of InSAR results for deformation.

**Fig 10:**
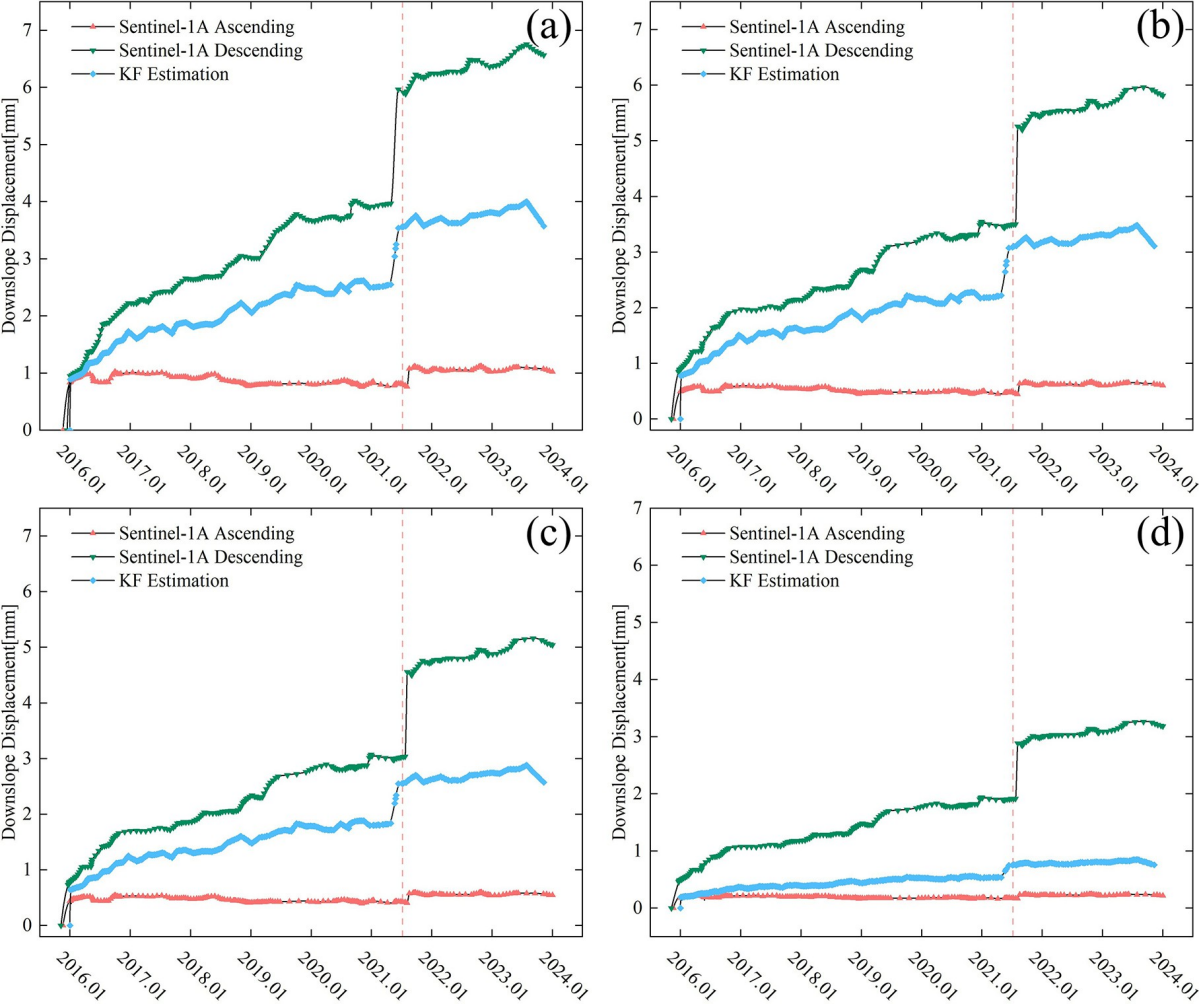
Downslope displacement and deformation for each track after Kalman filtering for different points of interest. (a-d) represent points of interest in the deformation region.

As shown in Table 1, the aspect of the landslide relative to true north is 193.4517 degrees. The displacement of the landslide is mainly distributed along the slope direction, which will lead to a decrease in the reliability of InSAR results in this direction. To further focus the spatiotemporal evolution of deformation characteristics on the landslide body itself, reduce the influence of different line-of-sight directions from radar platforms, and increase the reliability of time-series InSAR results, we projected the deformation along the LOS direction onto the slope direction and applied Kalman filtering to obtain the time-series deformation curve along the slope direction, as shown in Fig 10.

**Table 1:** Calculated parameters of the slope deformation transformation model at SAR images.

Platform	Heading Angle [degree]	Incidence Angle [degree]	Average Slope [degree]	Average Aspect [degree]
**Sentinel-1A Ascending**	-13.0961	33.7183	35.7566	193.4517
**Sentinel-1A Descending**	-166.9223	33.7858	35.7566	193.4517

In this paper, four points of interest were selected in the study area to explore the ability of Kalman filtered to fit downslope displacement. From the resulted in Fig 10, we can see that the deformation along the slope direction of the points on the ascending-descending orbit of the point of interest a has expanded from 0.82 mm and 0.94 mm to 1.02 mm and 6.51 mm. The maximum deformation in the slope direction of the points of interest b-d is about 3.8mm, 3.2mm and 0.7mm in that order. Although there are still differences in the InSAR deformation monitoring results from different radar orbits, they now exhibit stronger spatiotemporal consistency and have reduced the magnitude of differences. Since large-scale deformations occurring during the landslide phase can easily lead to the failure of the time-series InSAR detection technique, a dynamic iterative update of prediction-correction-prediction for the entire landslide displacement over time is conducted by fusing the ascending-descending time-series results from Sentinel-1A and the time-series InSAR results before and after the landslide through Kalman filtering. The filtered series curve is shown as the blue curve in Fig 10 Clearly, the inclusion of multi-platform detection data has enhanced the temporal resolution of time-series InSAR observations, reducing the shortest monitoring interval from a fixed 12-day observation cycle to 6 days. The filtered time series curve shows significant spatiotemporal consistency with the ascending-descending orbit monitoring results. During the filtering process, the covariance matrix was dynamically updated due to the correction of the data, and the slope deformations of points of interest a-d for the second landslide in 2021 were corrected to 3.2 mm, 2.9 mm, 2.4 mm, and 0.7 mm, in turn, by integrating the different observed features. Additionally, the supplemented time-series InSAR results of the slope displacement before and after the abrupt change provide an extra observational perspective for the InSAR time series detection of the landslide process, significantly enhanced the stability of landslide monitoring on different slope aspects and the results of large-scale abrupt changes.

## Discussion

### Deformation analysis results

**Fig 11:**
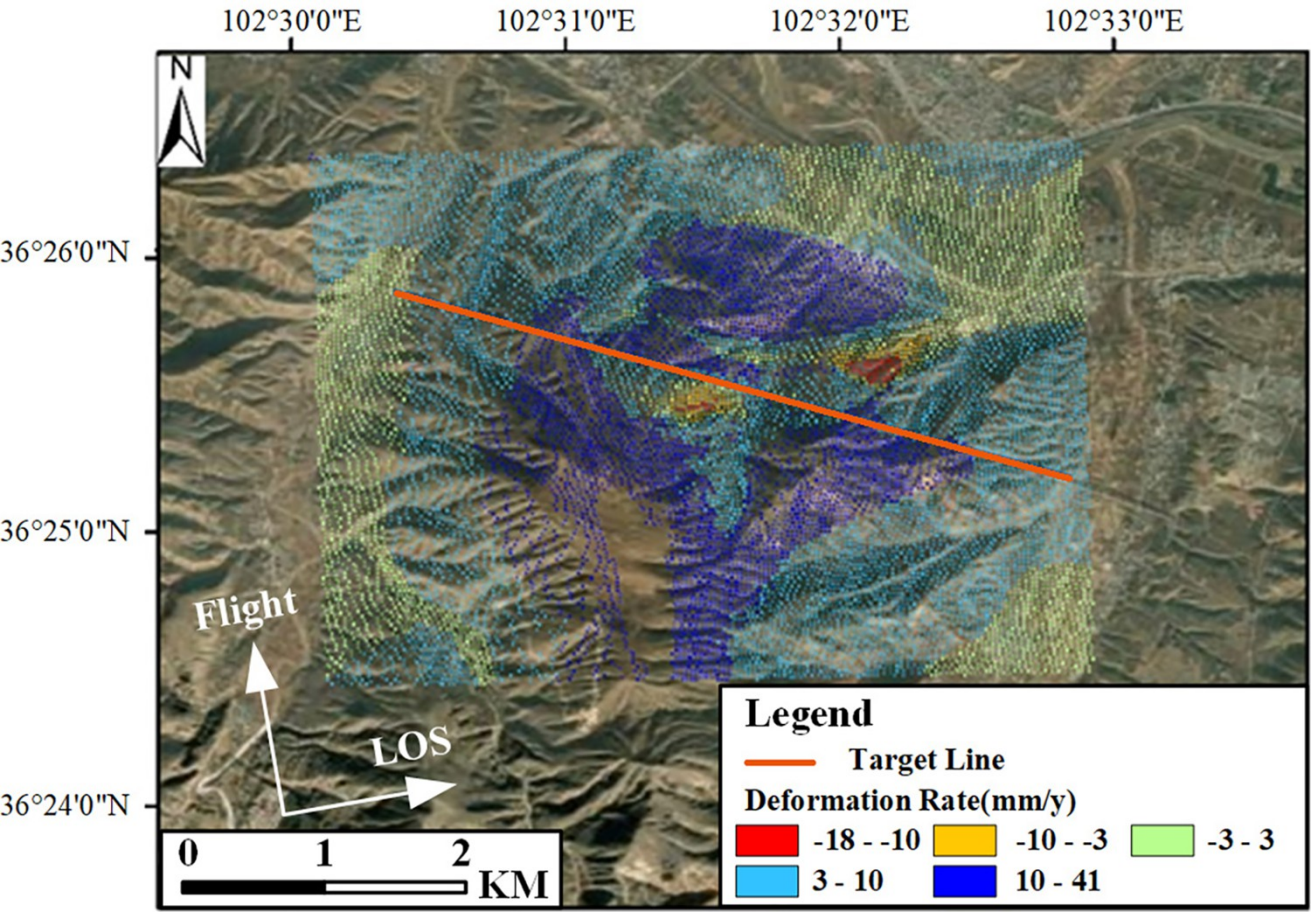
LOS average annual deformation rate map for the target area. Image basemaps and topography appearing in the text are from ESRI (www.ESRI.com).

Based on Sentinel-1 data and MT-InSAR technology, and using optical remote sensing imagery as the base map, the LOS average annual deformation rate map for the Gaojiawan landslide area was obtained, as shown in Fig 11.

In Fig 11, negative values indicate deformation moving away from the satellite sensor along the LOS direction, while positive values indicate deformation moving towards the satellite sensor along the LOS direction. The figure reveals that, during the monitoring period from December 17, 2020, to July 21, 2021, the maximum rate of movement away from the satellite sensor along the LOS direction in the target area was -18mm/year, and the maximum uplift rate was 41 mm/year. Deformation trends of sliding downward are present on both sides of the target route, especially in the red and yellow areas, which correspond to higher rates of sliding deformation and have a more significant impact on the health status of the route. Regarding the extracted deformation points, they are evenly distributed, except for a small blank area far south of the route. Overall, the density of points is sufficient to support detailed analysis of the landslide mass. For areas lacking deformation results, optical images reveal that the corresponding land types are mainly cultivated land, leading to severe decorrelation phenomena. The exclusion of these points from the deformation settlement ensures the reliability of the deformation results.

### Landslide deformation feature analysis

**Fig 12:**
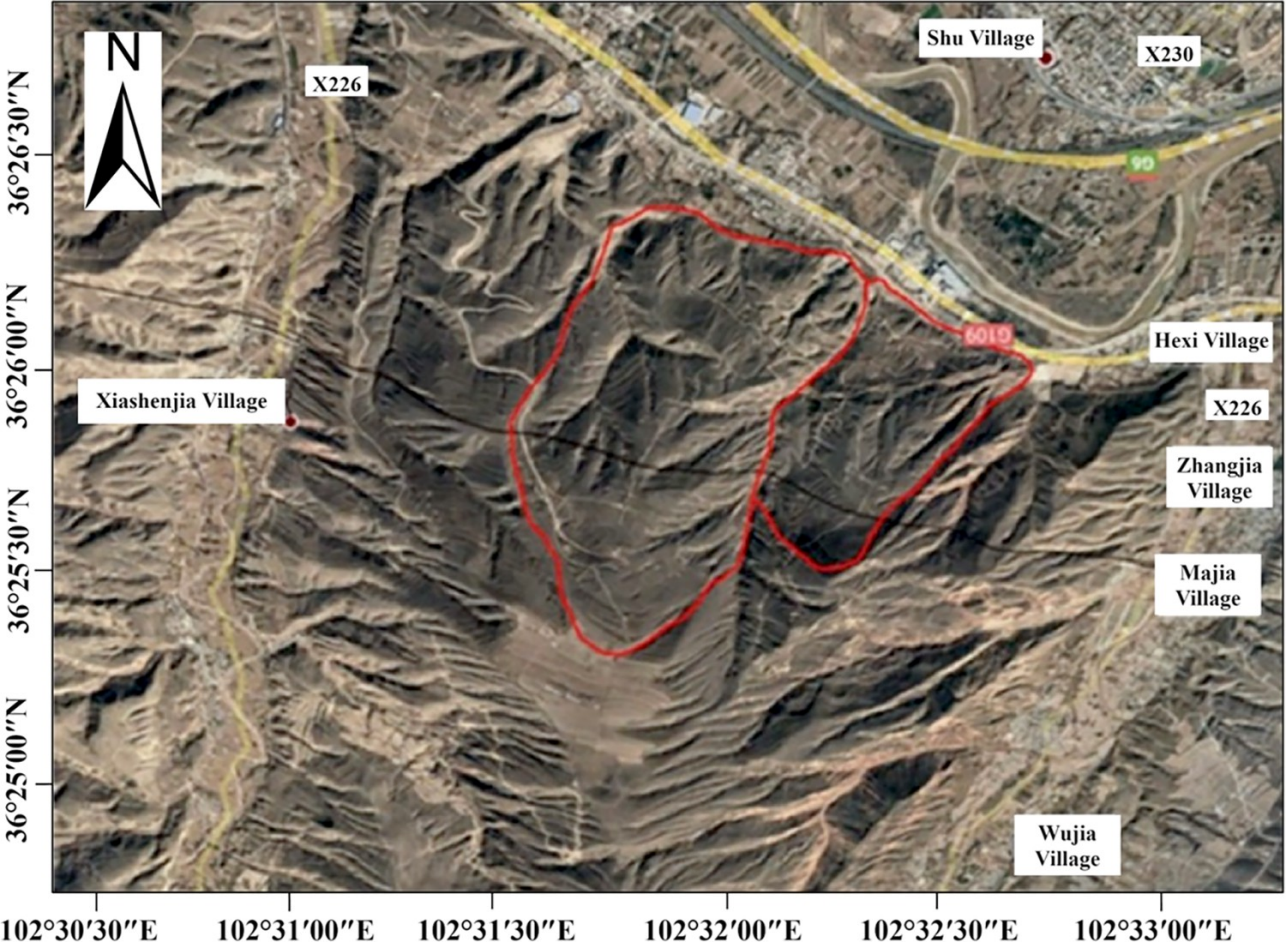
Boundary of the landslide mass. Image basemaps and topography appearing in the text are from ESRI (www.ESRI.com).

Initially, the development of the landslide mass was analyzed based on optical images, with the landslide’s boundary outlined based on morphological distribution characteristics as seen in the optical images, the results of which are illustrated in Fig 12.

**Fig 13:**
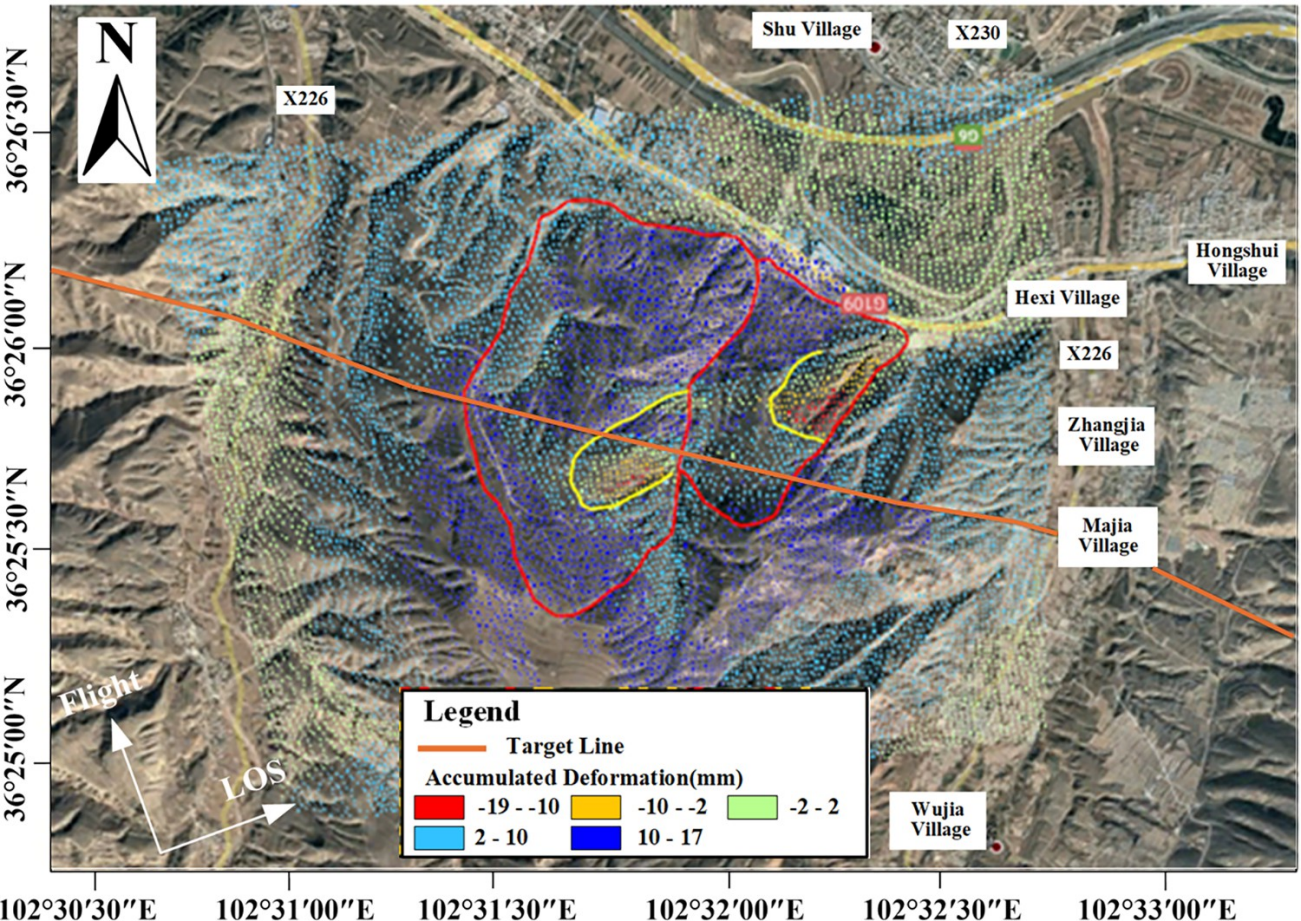
Overlay map of deformation rate results and landslide boundary. Image basemaps and topography appearing in the text are from ESRI (www.ESRI.com).

In Fig 12, based on the terrain and geomorphology, it can be observed that the main direction of the landslide mass is northeast. The landslide can be divided into eastern and western parts, with a distinct gully in the middle. The deformation rate results are overlaid on it, as shown in Fig 13.

**Fig 14:**
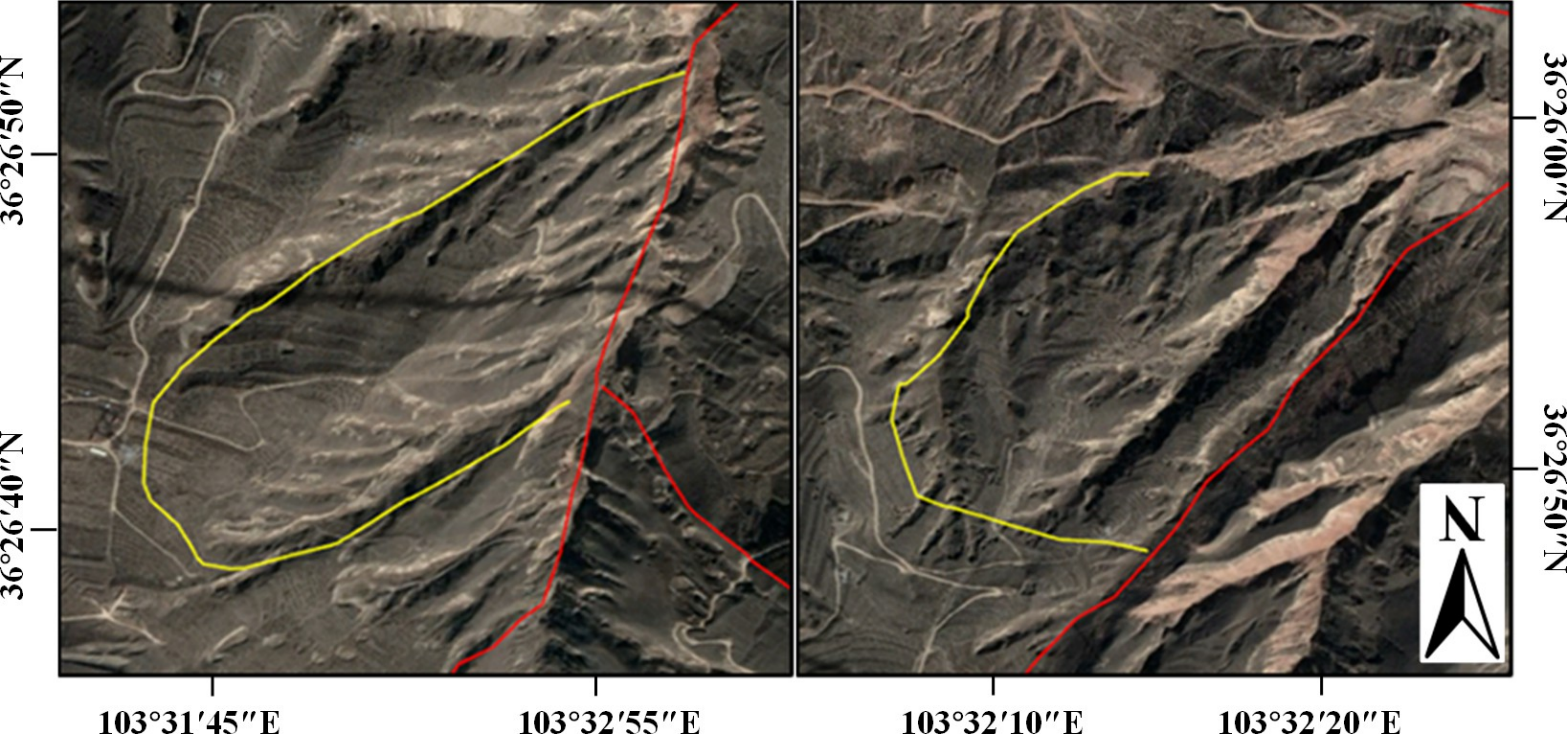
Morphology of sub-landslides. (Visible sliding area 1 on the left, visible sliding area 2 on the right). Image basemaps and topography appearing in the text are from ESRI (www.ESRI.com).

From Fig 13, it is evident that the front part is a large uplift area (along the ascending LOS direction), indicating that the landslide mass shows significant antiform bending at the landslide forefront, with a clear antiform shear exit observable on-site. In the middle part, multiple bands of red, blue, and green are visible, reflecting multi-level sliding in the surface along the LOS direction, with different sliding directions. Moreover, there is a clear positive (blue) and negative (red) rate division line in this landslide area, indicating opposite movements along the ascending LOS direction on both sides of the line. There are two significant sliding areas on the landslide mass (areas circled in yellow in Fig 13), which, when magnified and compared with optical images, are found to be two sub-landslides. The deformation rate result areas match well with the positions of the landslide body, as specifically shown in Fig 14.

For the sub-landslide on the left side in Fig 14, the deformation rate at the rear edge of the slope is higher, and the front edge is more stable, with the deformation rate decreasing along the slope direction, belonging to a rear-edge-pushing type; for the sub-landslide on the right side, located directly above the route, the deformation rate map reveals clear differences in deformation rates on both sides of the line. The south side mainly shows a downward sliding trend, the middle part has a smaller deformation rate and is more stable, while the north side shows a clear uplift trend. This distribution of deformation suggests that the route has absorbed most of the sliding energy from the south side, preventing significant deformation in the shallow part of the middle section, while the front edge shows clear uplift. This misaligned sliding greatly endangers the route, with the tunnel structures at the edge of the area showing significant cracking and block dropping after sliding in 2016 and 2021, proving the correctness of this inference.

**Fig 15:**
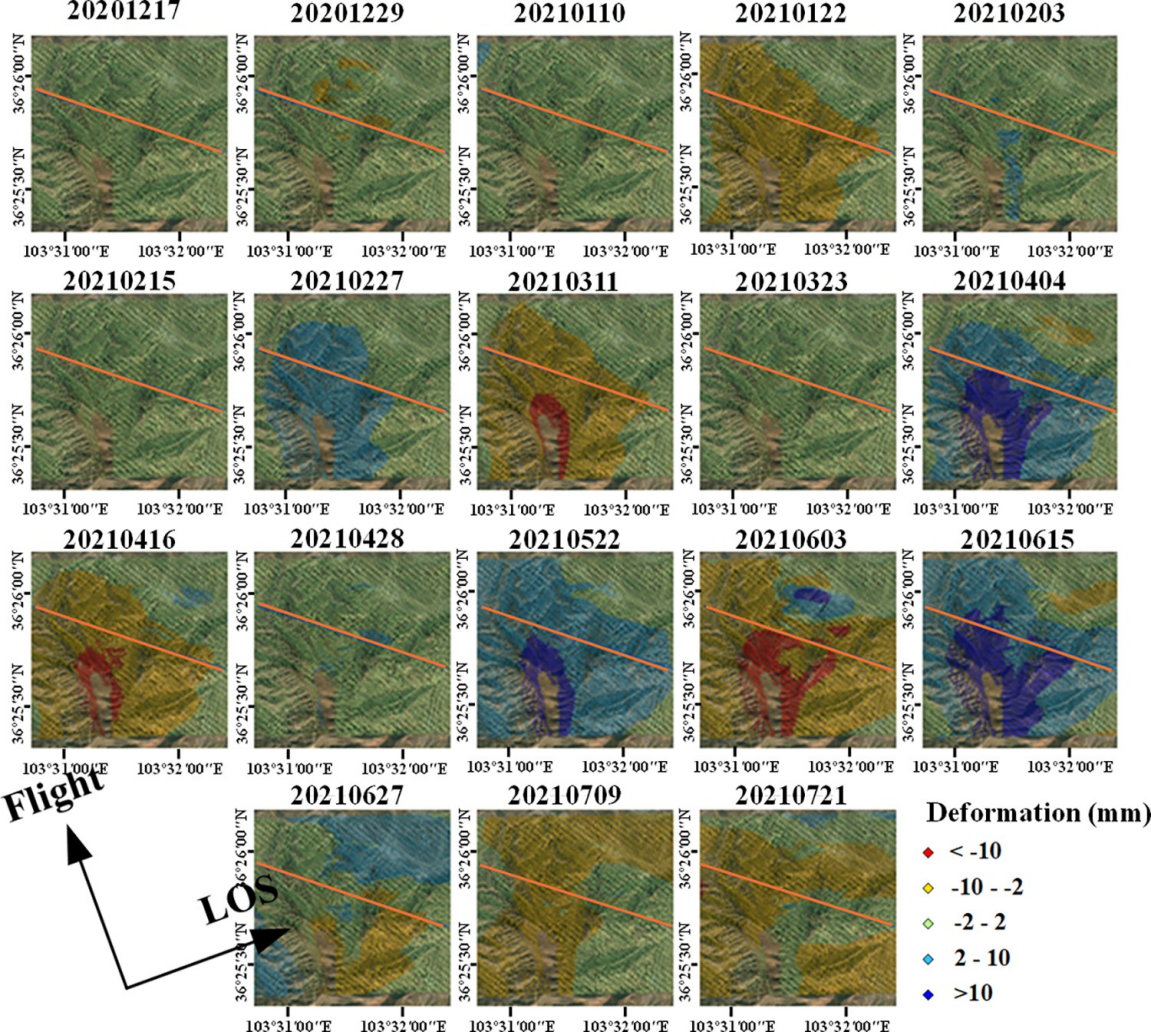
Time series of changes in the target area during the monitoring period from December 2020 to July 2021. Image basemaps and topography appearing in the text are from ESRI (www.ESRI.com).

Since the InSAR deformation rate represents the average spatial deformation information during the monitoring period and is mainly based on time-series deformation fitting, it does not fully reflect the temporal variation of deformation. To better leverage the temporal advantage of InSAR technology and showcase the deformation trend in the landslide area, we further analyzed the deformation condition in this area by combining time-series deformation data from different dates. Traditional time-series deformation information shows the cumulative deformation in the LOS direction within the monitoring period, representing the cumulative deformation of each observation date relative to the start date. For a clearer display of deformation changes between different dates, the cumulative deformation of two adjacent periods was subtracted, resulting in the change between neighboring dates, as shown in Fig 15.

**Fig 16:**
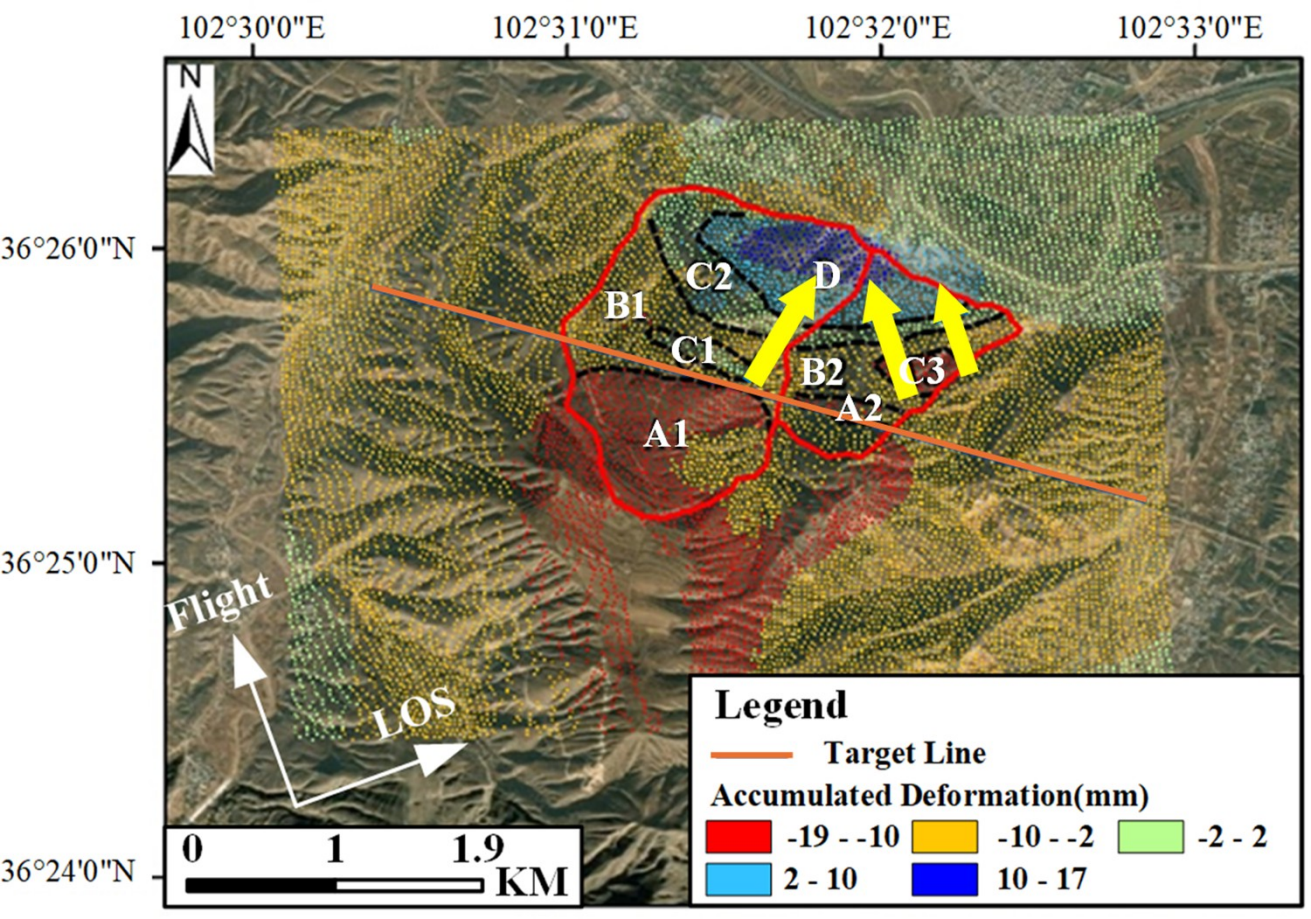
Deformation change map on June 3, 2021. Image basemaps and topography appearing in the text are from ESRI (www.ESRI.com).

From the time-series cumulative deformation change map, it can be seen that on March 11, 2021, April 4, 2021, April 16, 2021, May 22, 2021, June 3, 2021, and June 15, 2021, the southern part of the study area exhibited deformation with absolute magnitude exceeding 10mm, indicating active deformation in this part of the slope, especially on June 3, 2021, when different parts of the entire study area showed opposite trends of sliding and lifting, consistent with the distribution of landslides, while the deformation direction was generally consistent on other dates. On March 11, 2021, and April 16, 2021, sliding phenomena occurred on the southern side of the study area, likely related to the large-scale sliding on June 3, 2021. After June 15, 2021, the change map showed widespread, smaller magnitude deformations, which, combined with the objective sliding rules of the landslide and the principle of InSAR phase information composition, are speculated to be related to atmospheric effects. Significant sliding and lifting parts appeared in the study area between June 3, 2021, and the previous observation date, especially with clear differences in deformation on both sides of the route, and this date coincides with the deformation occurrence date of the target route. Therefore, this period’s deformation change is analyzed in detail to further elucidate the spatial distribution of the landslide, as shown in Fig 16.

Based on the magnitude of deformation changes during this period, the landslide area outlined by optical images has been divided into several sub-areas with different levels and directions of deformation. Areas A1, A2, and A3 are regions with significant downward sliding, primarily located at the top of the mountain, with the maximum change amounts reaching -19mm, -12mm, and -18mm, respectively. Moreover, areas A1 and A3 overlap with the positions of the two sub-landslides mentioned previously. Areas B1 and B2 exhibit slightly smaller levels of downward sliding, with maximum changes of -9mm and -9.9mm, respectively. Areas C1 and C2 are relatively stable, with deformation amounts ranging from -2mm to 2mm; Area D mainly encompasses uplift areas, primarily located at the foot of the mountain, with the maximum deformation amount being 17mm. For areas with significant downward sliding, given their position on the slope and using the slope deformation rate conversion formula mentioned earlier, where θ is 39.14°, α_s is -13.11°, and α and φ are set based on DEM data analysis at 40° and 15° respectively, the c value is approximately 0.696. Therefore, the maximum slope-directed sliding amounts for A1, A2, and A3 are approximately -27.3mm, -17.2mm, and -25.9mm, respectively, bringing these results closer to the actual deformation levels in the area.

**Fig 17:**
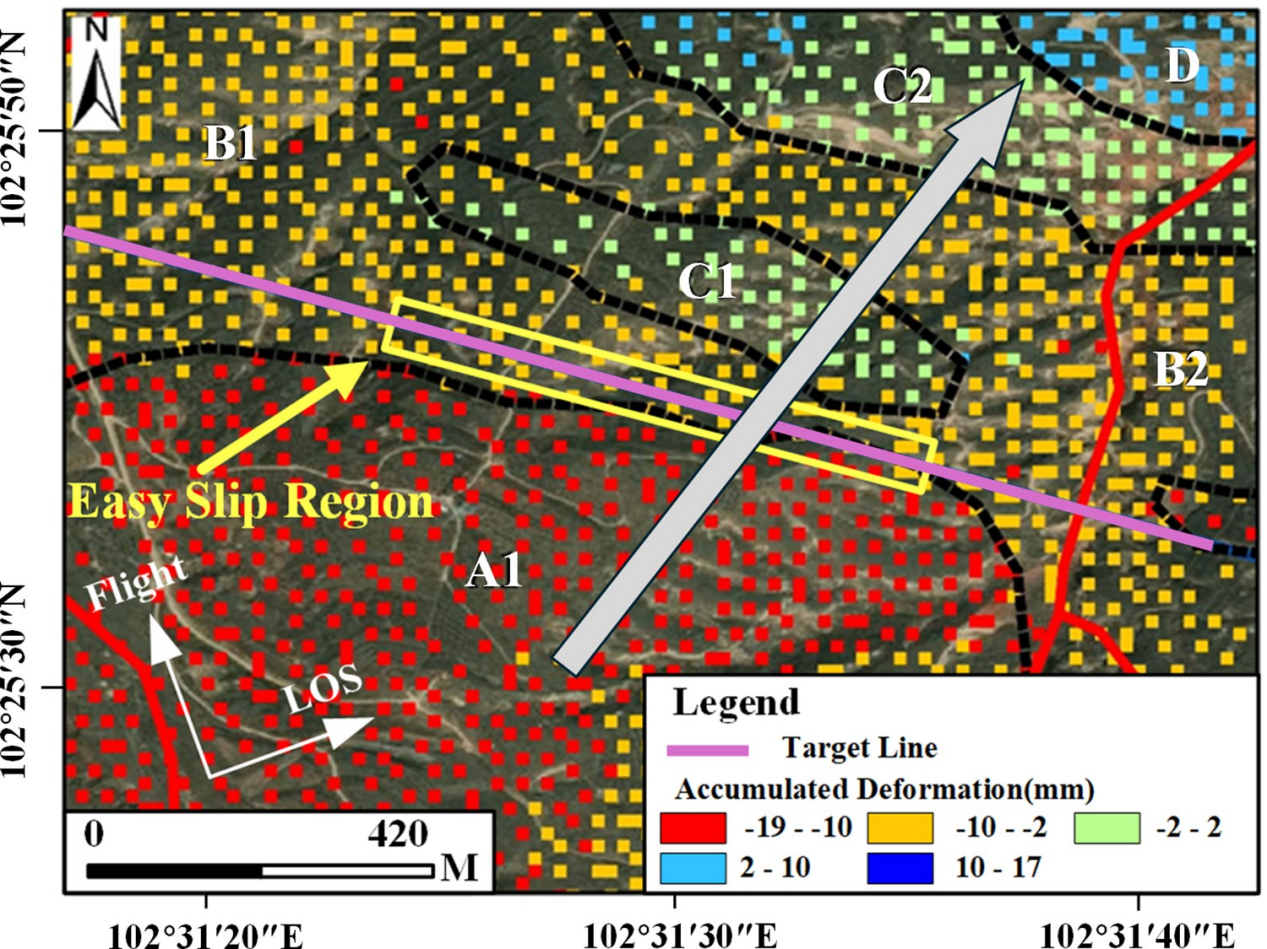
Distribution map of deformation features in key areas image basemaps and topography appearing in the text are from ESRI (www.ESRI.com).

Given that the different levels of deformation sub-areas are distributed in a step-like fashion, with areas of significant downward sliding mainly located at the rear edge of the slope and the front edge showing uplift, and the uplift area on the northwest side showing a deformation level significantly higher than the surrounding areas, it is considered that the landslide is a translational slide. The uplift at the front edge is primarily due to the anti-form bending of the landslide body’s resistance sliding surface at the sliding front, caused by shearing from the front edge outlet. Considering the distribution of deformation in this area, the sliding direction of the landslide body is indicated by the arrows in Fig 1. Additionally, it’s important to note that Area A1 is located to the southwest of the route, while Areas A2 and A3 are to the northeast. Due to the differences in deformation levels and the presence of a clear dividing line between them, the shear stress generated by such misaligned deformation poses a great risk to the route. Also, from the perspective of the route’s impact, combining the conclusions drawn from the analysis of deformation rate results earlier, the north side of the route in the C1 area is relatively stable, while the south side in the A1 area shows significant deformation levels. Considering the stress endured by the route and its impact on the route’s structure, this section is prone to misalignment, hence the need for focused monitoring. In the engineering mitigation process, the route’s structure in this position also needs to be significantly reinforced, as specifically illustrated in Fig 17.

Overall, the analysis indicates that the landslide body in this area is mainly influenced by pushing from the rear edge, leading to varying degrees of sliding in the middle part, while the front edge was relatively stable initially but showed an uplift deformation trend under the influence of the rear edge push later on. It is also noteworthy that within the monitoring period, the area was relatively stable from the start date until March 11, 2020, after which the deformation magnitude began to increase. This information can serve as a reference for issuing warnings and focusing monitoring efforts on geological disasters.

### Model applicability analysis

Due to the geometric differences in satellite imaging between ascending and descending orbits, significant discrepancies are observed in the landslide deformation results. Both ascending and descending orbits provide deformation information in the Line-of-Sight (LOS) direction, rather than in the slope direction. For example, the deformation and phase results from the ascending and descending orbits in Figs 5 and 7 fail to fully represent the landslide deformation characteristics. Therefore, it is crucial to fuse ascending and descending orbit InSAR data for slope-direction deformation extraction in order to achieve precise monitoring of landslide areas. Previous studies have completed deformation monitoring and analysis of the Gaijiawan landslide in the same region using ascending and descending orbit InSAR, but they have focused solely on LOS direction deformation, rather than slope-direction deformation [46]. This study, however, utilizes a fusion of ascending and descending orbits to extract slope-direction deformation information, revealing local deformation characteristics within the landslide body. By further analyzing the landslide’s dynamic process, this approach provides a more reliable basis for early warning and risk assessment of landslides.

In terms of the application of the Kalman filtering model, similar studies have also explored and analyzed this approach. These studies applied the Kalman filter to a multi-sensor, multi-orbit, multi-temporal InSAR method for analyzing three-dimensional surface displacement [47]. The results indicate that this method not only determines three-dimensional displacement rates but also tracks the temporal evolution of displacements. An important feature of this method is its ability to combine measurement data from different radar sensors, which improves the temporal resolution of measurements. This demonstrates the feasibility of applying Kalman filtering to multi-orbit data fusion in InSAR.

For model fusion methods, including machine learning and Bayesian approaches, although they have certain advantages, they still have limitations. For instance, the performance of machine learning methods is closely related to the amount of sample data, and they are sensitive to small sample sizes and noise. Moreover, their models often lack interpretability, making it difficult to provide clear physical meanings [48]. Bayesian methods, on the other hand, require high computational complexity when processing large-scale data and necessitate careful selection of prior distributions. Additionally, they may not reflect deformation processes in real-time when there are rapid dynamic changes. In contrast, Kalman filtering does not require training samples or predictive data, and it can directly estimate deformation. Furthermore, this method can achieve more accurate dynamic monitoring of the landslide body by integrating data from multiple platforms, orbits, and time series. The fusion model proposed in this study significantly compensates for the limitations of satellite-based SAR in landslide monitoring, particularly in terms of temporal resolution, making it highly applicable and valuable for practical use.

## Conclusions

This study addresses the limitations in accurately reflecting the true deformation of landslides and the low temporal resolution of landslide monitoring, which hinders long-term dynamic reconstruction. We propose a method for dynamic reconstruction and evolutionary feature analysis of slope deformation using Kalman filtering based on ascending and descending track time-series InSAR observations for the Gaojiawan landslide in the Lanzhou-Xinjiang Railway Tunnel. This method integrates MT-InSAR and D-InSAR techniques to extract time-series deformation data by selecting coherent points in the study area. The LOS deformation of the landslide is then converted into slope deformation, and Kalman filtering is employed for dynamic reconstruction of the landslide deformation. Experimental results demonstrate that the proposed method enhances the temporal resolution of Sentinel-1A satellite monitoring to 6 days. Furthermore, after Kalman filtering and merging the ascending and descending track slope deformations, the cumulative slope deformation for the first landslide event in January 2016 is 2.38 cm, and for the second event in June 2021, it is 3.9 centimeters, with other periods showing relative stability. This approach significantly improves the reliability of landslide monitoring post integration of ascending and descending track deformations, providing crucial technical methods and data support for disaster prevention and mitigation in similar high-risk geological hazard areas.
